# Empirical insights into industrial policy’s influence on phytoprotection innovation

**DOI:** 10.3389/fpls.2023.1295320

**Published:** 2023-11-14

**Authors:** Yangxun Zhang, Shaoqiang Li, Fengyun Wu

**Affiliations:** ^1^ School of Finance and Economics, Guangdong Polytechnic Normal University, Guangzhou, China; ^2^ School of Modern Information Industry, Guangzhou College of Commerce, Guangzhou, China

**Keywords:** intelligent phytoprotection, industrial policy, patent, DID, empirical view

## Abstract

Intelligent Phytoprotection is an important direction for the modern development of plant protection related disciplines, and its essence is the innovative application of new generation information technology industry, high-end equipment manufacturing industry, and digital industry related technologies in the traditional plant protection field. This article first identifies 224 International Patent Classification (IPC) Main groups in the field of intelligent phytoprotection technology based on the International Patent Classification System. And then combines with China’s industrial policy practice, we explore the impact of industrial policy on the application number of invention patents in the field of intelligent phytoprotection technology using the Difference-in-difference (DID) method and the Synthetic DID method. The study results showed that the implementation of industrial policy can significantly promote the patent application activities in the intelligent phytoprotection treatment group, with an average increase of 517 invention patent applications compared to the control group that is not affected by the policy. The research conclusion of this article suggests that for countries and regions, industrial policies are an important tool for promoting the innovation and development of intelligent phytoprotection related technologies.

## Introduction

Intelligent phytoprotection is a comprehensive technology system based on computer vision, internet and cloud computing, big data, artificial intelligence, satellite, and global positioning, and using computers, smartphones, drones, and various agricultural machinery or equipment as terminals for monitoring, early warning, and management of plant diseases and pests (Huang and Shu, 2021; Wang, 2022). It is a comprehensive science that integrates plant protection with multiple disciplines. As a key component of smart agriculture, the development of intelligent phytoprotection and its related technologies has attracted the attention of countries around the world.

Intelligent phytoprotection constitutes a comprehensive technological system integrating computer vision, internet and cloud computing, big data, artificial intelligence, satellite technology, and global positioning. It employs computers, smartphones, drones, and various agricultural machinery as monitoring and management terminals for the early detection and control of plant diseases and pests (Huang and Shu, 2021; Wang, 2022). It represents a multidisciplinary science, tightly interwoven with plant protection, and holds a central role in the realm of smart agriculture. Nations worldwide have been increasingly focusing their attention on the development of intelligent phytoprotection and its associated technologies.

As an emerging industry, the development of innovative activities in related fields of intelligent phytoprotection may be affected by various factors, among which industrial policy is the most important one. Specifically, the policy toolkit, including financial support and tax incentives, contained in industrial policy can guide resources to concentrate on intelligent phytoprotection-related technology fields, and to a certain extent, eliminate non-economic obstacles faced by enterprises in carrying out innovative activities in new fields, thus promoting the development of intelligent phytoprotection innovation activities. In a world marked by deepening globalization and relentless technological advancement, the swift exchange of knowledge and technology between countries has led developed nations to enact policies such as trade protection, restrictions on the movement of scientific and technological personnel, and measures to encourage the repatriation of manufacturing in order to safeguard their technological dominance. Concurrently, environmental concerns, notably climate change, are compelling nations to revamp antiquated, energy-intensive, and polluting industries. These shifts in objective realities have galvanized both developed and developing countries to explore the deployment of industrial policies as catalysts for their own technological advancement and innovation (Naudé, 2010; Salahuddin and Yülek, 2022).

In recent years, academic literature has increasingly focused on the important role of industrial policies in nurturing emerging industries and promoting developing countries to leapfrog the middle-income trap. Various perspectives have been explored, including stimulating the learning process of local enterprises, alleviating negative externalities, and promoting the efficient transfer of domestic labor from the agricultural sector to the knowledge-based manufacturing sector, revealing the potential of industrial policies as a driving force for technological progress (Stiglitz and Greenwald, 2014; Stiglitz, 2017; Yülek, 2018). This article subscribes to the notion that industrial policy can indeed propel the development of innovative technologies. The distinction between intelligent phytoprotection and conventional plant protection technologies hinges on their close integration and deep synergy with emerging industries, particularly the information technology sector, high-end equipment manufacturing, and the digital economy – areas of paramount focus in the formulation of national industrial policy development plans by most countries. For example, Japan’s Ministry of Economy, Trade, and Industry, in its Industrial Structure Vision 2010, proposed the formulation of industrial policies to bolster high-end equipment manufacturing and information technology industries (The Ministry of Economy, Trade, and Industry of Japan, 2010). Similarly, the European Commission (2012) advocated for prioritizing key enabling technologies (such as semiconductor technology, biotechnology, and optoelectronic material manufacturing) and bioproduct manufacturing (including biodegradable textile fibers and biofuels produced from bioproducts) in its policy report titled “A Stronger European Industry for Growth and Economic Recovery” in 2012. China, in its 2010 strategic emerging industry policy, listed the new generation of information technology and high-end equipment manufacturing as strategic emerging industries deserving support and cultivation. In the policy revision of 2016, the digital economy industry was explicitly designated as a national-level strategic emerging industry.

For the advancement of the intelligent phytoprotection industry and its associated technologies, industrial policy emerges as a pivotal determinant. This study leverages global granted invention patent data from the Clarivate PLC’s incoPat patent database to identify intelligent phytoprotection patents, extract their technical attributes, and establish a classification of intelligent phytoprotection-related patents at the International Patent Classification (IPC) Main Group level. Furthermore, we pinpoint intelligent phytoprotection-related invention patent data originating from China based on technological characteristics and geographical location information. Simultaneously, by scrutinizing China’s 11th,12th,13th and 14th Five Year Plan documents and conducting text analysis, we identify the Strategic Emerging Industrial Policy as the key governmental industrial policy document relating to the development of intelligent phytoprotection. Through an in-depth examination of these industrial policy documents and patent application data pertaining to intelligent phytoprotection inventions, we endeavor to unveil the influence of industrial policy implementation on patent application behaviors in the domains of intelligent phytoprotection-related technologies. In conclusion, we reflect on the implications of our research findings for various stakeholders, both within the industrial and academic realms, and suggest strategies for shaping their research and development initiatives in the field of intelligent plant protection, within the broader context of pertinent industrial policy planning and execution.

## From traditional plant protection to intelligent phytoprotection: a comparative analysis of innovation approaches

In contrast to conventional plant protection methods, which employ fertilizers, pesticides, machinery, and similar means for preventing diseases and pests while safeguarding plant health, intelligent phytoprotection relies predominantly on information technology. This approach leverages modern Internet-based technologies such as drones, plant protection robots, and intelligent insecticidal lighting systems to accomplish its plant protection objectives. Wang (2022) has succinctly outlined the core technologies underpinning intelligent plant protection, emphasizing their utilization of contemporary information technology. These technologies encompass Sensor technology, Computer Vision technology, Image Processing technology, 3S technology, Internet of Things (IoT) technology, Intelligent Agricultural Machinery technology, Modern Network Communication technology, Big Data technology, Cloud Computing technology, and Artificial Intelligence technology.

Sensor technology, serving as the foundational hardware for smart plant protection, plays a pivotal role in addressing pest and disease occurrences, natural environmental fluctuations, and other related factors (Roshan et al., 2022). Computer vision technology and image processing technology provide essential support to intelligent plant protection by enabling automatic identification, detection of pests and diseases, and remote diagnosis, assessment, and monitoring of damage severity (Qin et al., 2016; Oppenheim et al., 2019; Singh et al., 2021). 3S technology equips intelligent plant protection with the capability to gather, process, analyze, and manage specific spatial and environmental information, enabling predictions and evaluations of pest and disease issues primarily influenced by environmental factors (Sishodia et al., 2020; Wang et al., 2021). IoT technology primarily offers real-time monitoring of plant growth, encompassing external environmental conditions, growth progress, pest and disease occurrences, and other pertinent factors (Abbasi et al., 2019; Phasinam et al., 2022a). The integration of intelligent agricultural machinery, such as drones and robots, into plant protection has facilitated the transition from traditional approaches to smart plant protection, ushering in mechanization, automation, precision, and intelligence in plant protection operations (Chin et al., 2023). Modern network communication technologies, exemplified by Internet Protocol Version 6 (IPv6), serve as the infrastructure of smart agriculture and play a pivotal role in ensuring the efficiency of information transmission and sharing among various equipment types within smart plant protection (Avsar et al., 2021). Big data and cloud computing technologies provide crucial support to smart agriculture by offering storage solutions and enabling distributed sharing of computing resources (Kumar, 2020; Rao & Yuan, 2021; Phasinam et al., 2022b; Rathod et al., 2022). Artificial intelligence technology delivers vital technical support for intelligent plant protection in areas such as information recognition, automated decision-making based on data sets, and the automated training and optimization iterations during data processing (Benos et al., 2021; Li, 2021; Mohan et al., 2023).

**Figure 1:**
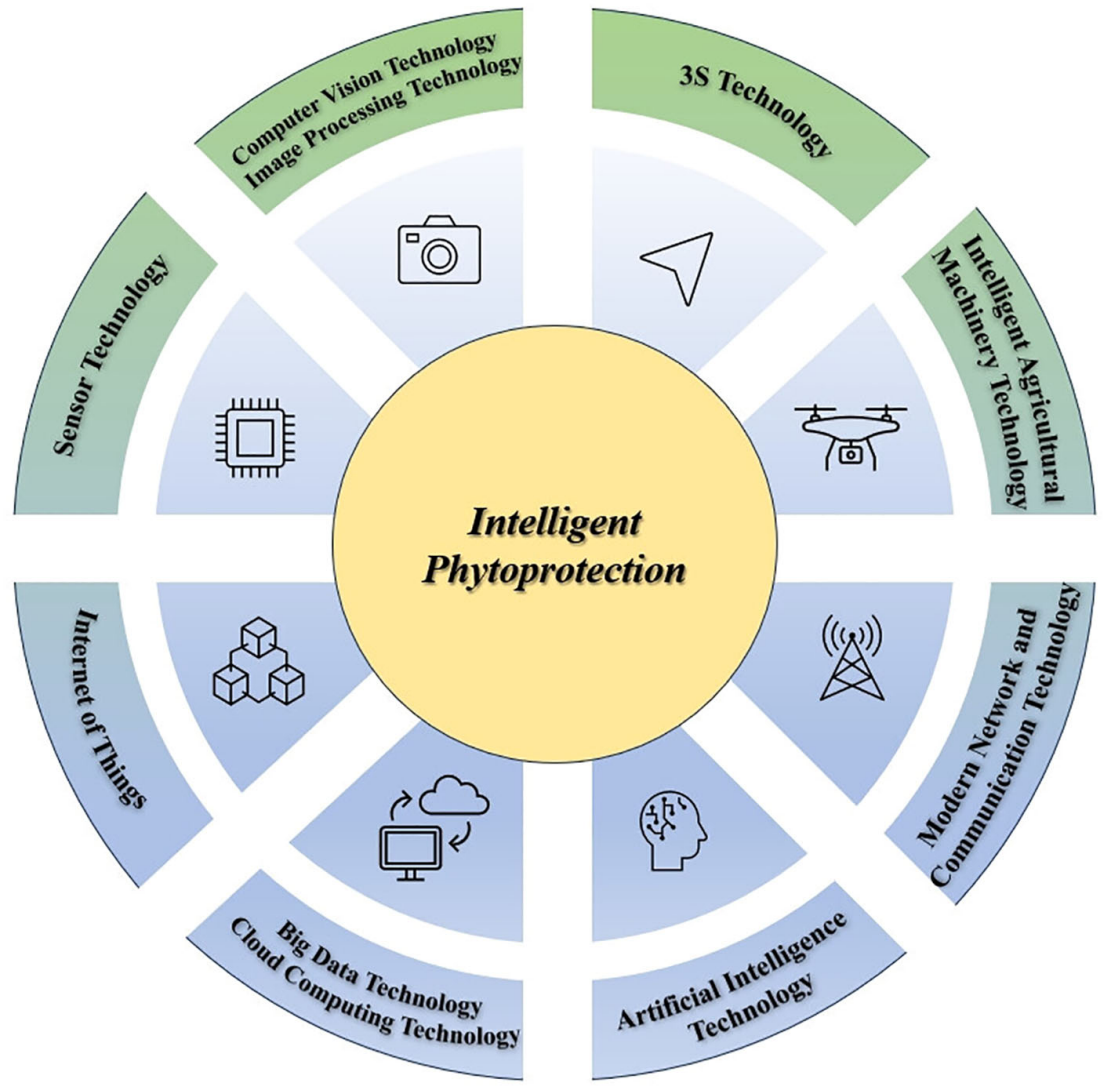
The key technologies of intelligent phytoprotection.

In comparison to traditional innovation endeavors in plant protection, innovation activities in intelligent phytoprotection exhibit a significantly higher degree of technical integration with emerging industry-related technologies, such as New Generation Information Technology, High-end Equipment Manufacturing, and Digital Creative Industries. Consequently, they demonstrate a notably informational, automated, and intelligent nature. The key technologies of intelligent phytoprotection are summarized in **Figure 1**.

Patent literature stands as the most effective repository of technical information, encapsulating over 90% of the latest global technological advancements. In our assessment of innovation activities, we have chosen patents as the primary yardstick and employed the incoPat patent database as our search tool. The incoPat global patent database encompasses data from more than 120 countries and organizations worldwide, representing a comprehensive repository managed by the global patent database service provider, Clarivate PLC.

**Figure 2:**
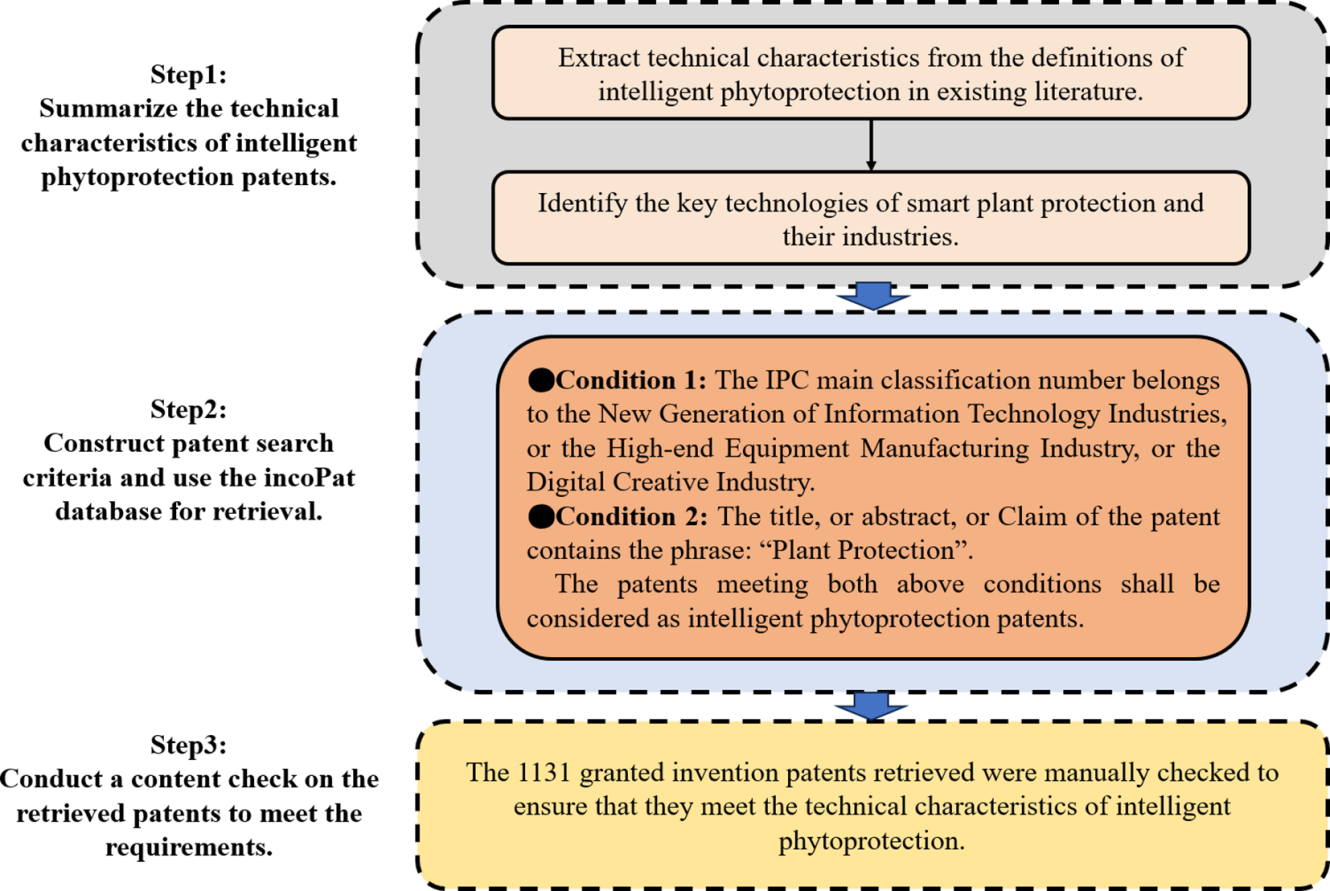
The schematic diagram of the patent retrieval process of intelligent phytoprotection.

Drawing upon the definitions of intelligent plant protection present in existing literature, we have identified patents within IPC main classification numbers related to the New Generation of the Information Technology Industry, High-end Equipment Manufacturing Industry, or Digital Creative Industry. These patents also contain the term “plant protection” in their titles, abstracts, or claims, signifying them as Intellectual Property Rights (IPR) associated with intelligent phytoprotection. Specifically, the New Generation of Information Technology industries encompasses Next-generation Information Network technology, Core Electronic technology, Emerging Software and New Information technology, Internet and Cloud Computing technology, Big Data Services technology, and Artificial Intelligence technology. The High-end equipment manufacturing industry includes Intelligent Manufacturing Equipment technology, Aviation Equipment technology, Satellite and Its Application technology, Rail Transportation Equipment technology, and Marine Engineering Equipment technology. The Digital Creative Industry encompasses Digital Creative Equipment Manufacturing, Digital Cultural Creative Activities, Design Services, and Digital Creative and Integration Services. We input these defined search criteria into the incoPat patent database for our patent search, ultimately yielding 1131 granted invention patents worldwide as of August 2023, with application years spanning from 2004 to 2023. The conditions and procedures governing the patent search are illustrated in **Figure 2**.

### Technical features of intelligent phytoprotection patents

**Figure 3:**
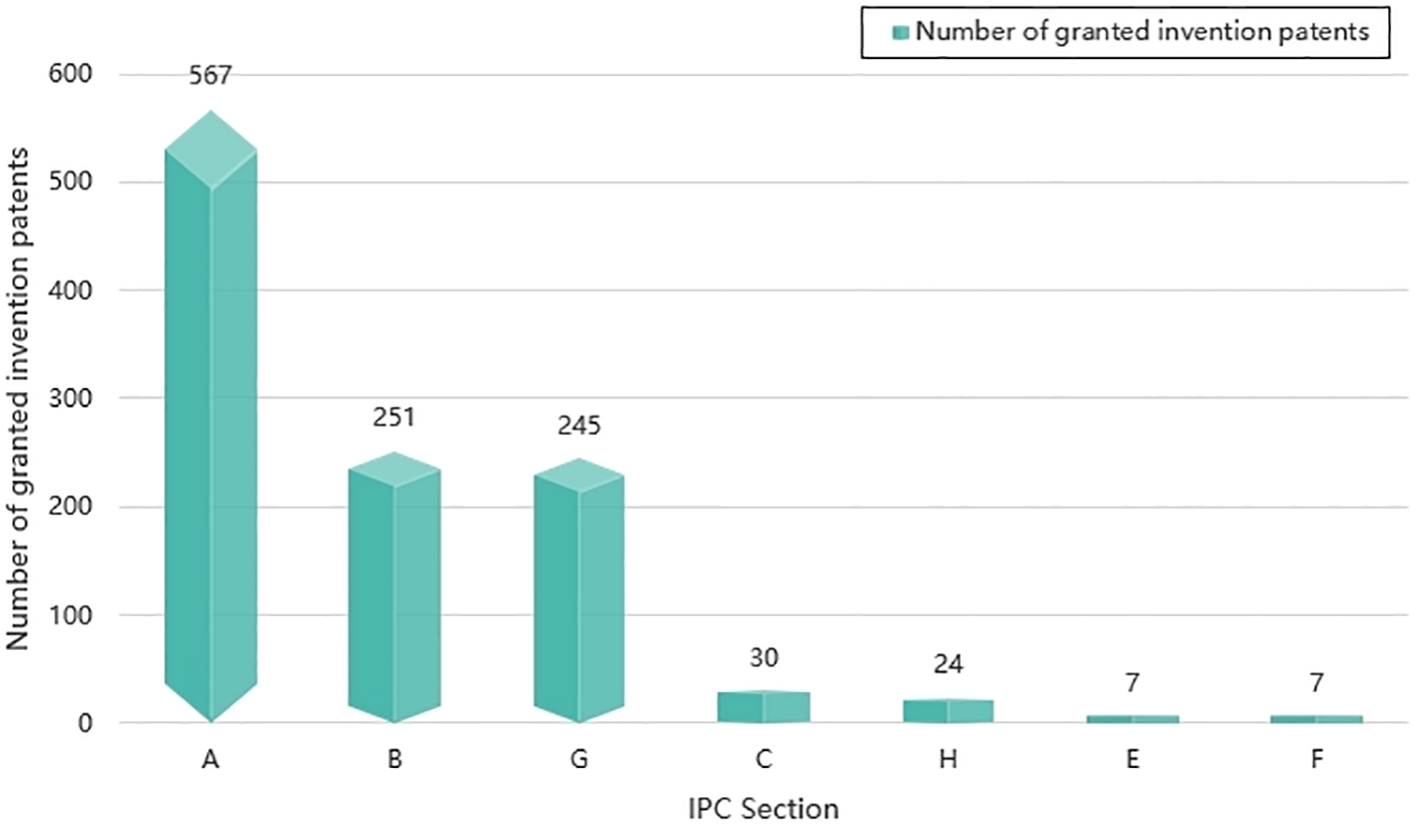
The proportion of intelligent phytoprotection patents in IPC main classification number (Section). Section (A) (Necessary for human life, including agriculture, forestry, etc.). Section (B) (Operation; Transportation). Section (G) (Physics). Section (C) (Chemistry; Metallurgy). (H) (Electricity). (E) (Fixed Construction). (F) (Mechanical engineering; Lighting; Heating; Weapons; Explosion).

Through the previously described patent search process, we have identified a total of 1,131 granted invention patents pertaining to intelligent phytoprotection on a global scale. These patents can be categorized into seven sections, and their distribution is visually represented in **Figure 3**. **Figure 3** illustrates that Section A constitutes the largest proportion, accounting for 50%. Following closely are Sections B and G, both at 22%. This distribution aligns with the fact that plant protection inherently falls within the domain of agriculture and forestry, making it consistent that the majority of granted invention patents related to intelligent phytoprotection reside in Section A. Sections B and G are primarily associated with unmanned aerial vehicle technology and automation control technology in the context of intelligent phytoprotection, hence their representation is in accordance with the technical characteristics of intelligent phytoprotection elucidated in existing literature.

Moreover, the five patent classes with the highest cumulative count of granted invention patents are A01 (agriculture, forestry, animal husbandry, hunting, trapping, and fishing), B64 (aircraft, aviation, and space navigation), G05 (control, regulation), G06 (calculation, projection, or counting), and B65 (transportation, packaging, and storage).

Out of the 1,131 granted invention patents, we further analyze the IPC main classification numbers at the IPC main group level, yielding a total of 224 IPC main groups. These 224 main groups span seven sections: A, B, C, E, F, G, and H. Section A (essential to human life) holds the highest proportion at 30.36%, followed by Section G (Physics) at 27.68%, and Section B (Operations; Transportation) at 23.21%. The IPC main groups with the most cumulative invention patents within the IPC main classification numbers are B64D1 (Throw, eject, release or receive items, liquids, or similar materials during flight), A01M7 (The special configuration or arrangement of liquid spraying equipment used to destroy harmful animals or plants), and G05D1 (The control of the position, path, altitude, or attitude of means of transportation on land, on water, in the air, or in space, such as an autopilot), predominantly pertaining to areas such as plant protection drones, drone flight control systems, and spraying systems.

**Figure 4:**
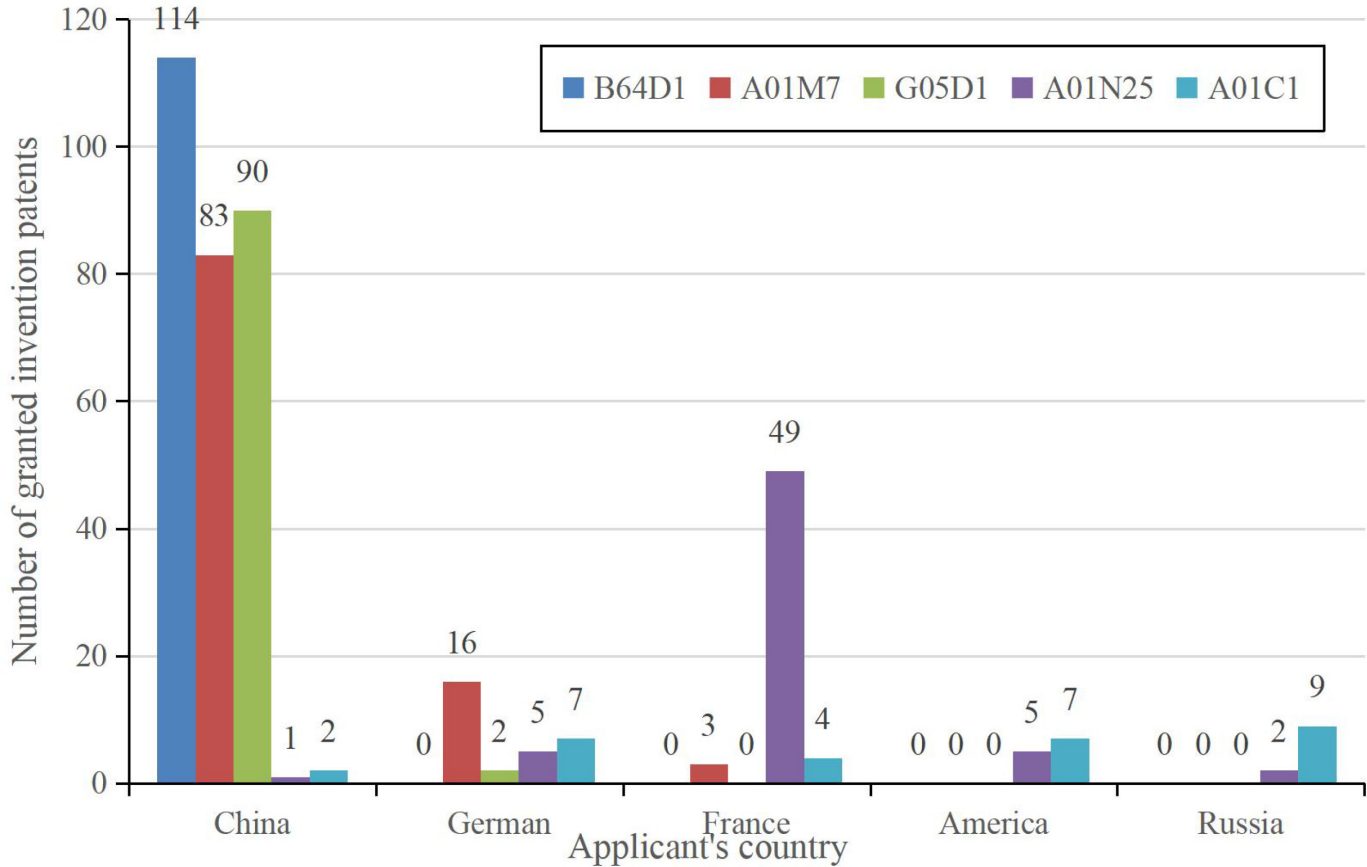
Top 5 IPC main groups and applicant’s countries within in terms of accumulated application numbers of granted inventions for intelligent phytoprotection.B64D1 (Throw, eject, release, or receive items, liquids, or similar materials during flight). A01M7(The special configuration or arrangement of liquid spraying equipment used to destroy harmful animals or plants). G05D1(The control of the position, path, altitude, or attitude of means of transportation on land, on water, in the air or in space, such as an autopilot). A01N25(Biocides, insect repellents or attractants, or plant growth regulators characterized by their morphology, non-active ingredients, or method of use; Substances used to reduce the harmful effects of active ingredients on organisms other than pests). A01C1(Equipment or methods for testing or treating seeds, roots, or the like before sowing or planting).

By amalgamating information about patent applicants’ countries and IPC main classification data, we have identified the country distribution of patent applicants for the top five IPC main groups in terms of cumulative granted invention patents for intelligent phytoprotection, which is visually represented in **Figure 4**. As illustrated in **Figure 4**, the top five countries in the world engaged in these activities are China, Germany, France, the United States, and Russia. Notably, China exhibits a competitive advantage in technological domains associated with B64D1, A01M7, and G05D1.

Based on the Web of Science (WOS) subject classification, the top three IPC main groups for cumulative patent applications are A1201 (Instrumentation), B0201 (Agronomy-Integrated Agronomy), and A0302 (Engineering-Electrical and Electronics). Furthermore, analyzing the frequency of technical efficiency phrases extracted from patent application abstracts reveals that major patent groups like B64D1 and A01M7, which rank highly in terms of cumulative patent applications, predominantly emphasize efficiency enhancement, convenience improvement, stability enhancement, safety enhancement, and automation enhancement. These technical efficiency characteristics, as conveyed in the abstracts, are well-aligned with the attributes of intelligent phytoprotection.

This section presents a comprehensive technical feature analysis based on the 1,131 authorized invention patents related to intelligent plant protection. It extracts 224 significant patent groups utilizing IPC primary classification information within the patent data. As previously mentioned, the essence of intelligent plant protection revolves around the application of innovative technologies like network communication, computer vision, and artificial intelligence within the field of plant protection. Consequently, we employ the 224 patent groups derived from intelligent plant protection patents to explore global invention patent application data further, shedding light on pertinent innovative activities in the realm of intelligent plant protection.

### Trends in invention patent applications for intelligent phytoprotection-related technologies

Building upon our previous analysis of the technical characteristics of granted invention patents in the field of intelligent phytoprotection, we utilized the 224 IPC main groups extracted from this analysis as the basis for IPC main classification numbers. Subsequently, we conducted a comprehensive search of the incoPat patent database to retrieve global invention patent application records filed from 2017 to 2021. This search yielded a substantial dataset of 3,429,252 invention patent application records.

**Figure 5:**
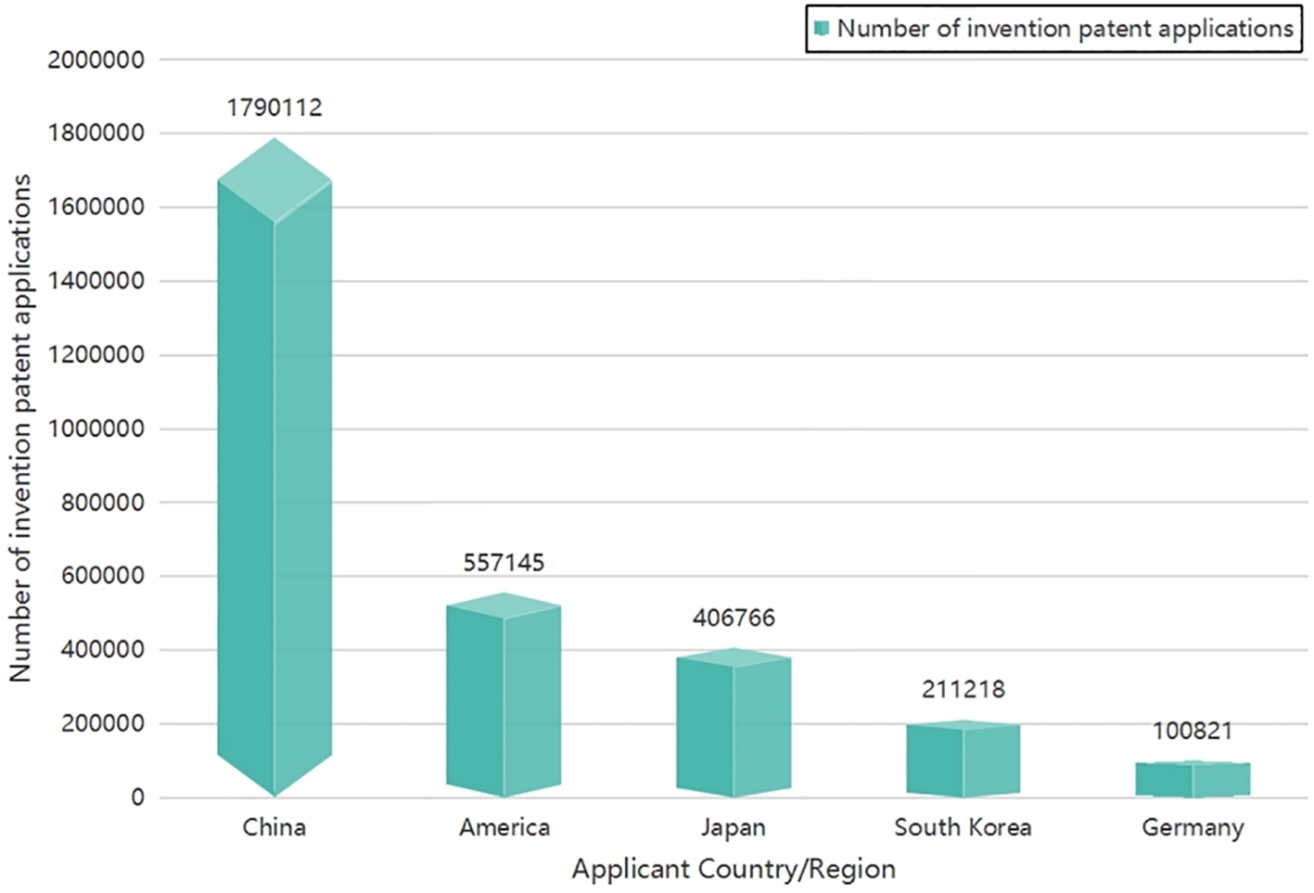
The top 5 countries in terms of the cumulative number of invention patents applied in the 224 IPC main groups of intelligent phytoprotection from 2017 to 2021.

By scrutinizing the applicant countries and regions and aggregating the cumulative number of invention patents applied for within this dataset, we present our findings in **Figure 5**. It is evident from **Figure 5** that over the span of five years, China held the foremost position in terms of the cumulative number of invention patents applied for within the domain of intelligent phytoprotection technology.

To gain deeper insights into the global technology landscape of intelligent phytoprotection invention patent applications, we conducted an in-depth examination of the technology classification information within the patent data. Specifically, we focused on the top ten IPC main groups with the highest number of invention patent applications originating from five prominent countries: China, the United States, Japan, South Korea, and Germany. These ten patent groups encompass a diverse array of technological domains:

1. G06F3 (Input means for transforming data for computer processing; output devices, such as interface devices used for data transmission from processors to output devices).
2. G06F16 (Information retrieval; database structure; file system structure).
3. G06Q10 (Administration; Management, including data processing methods, devices, and storage media).
4. G06K9 (Methods or devices for pattern recognition, involving the graphical interpretation or conversion of mechanical parameter patterns into electrical signals).
5. H01L21 (Methods or equipment specialized for semiconductor or solid device manufacturing or processing).
6. H04L29 (Devices, equipment, circuits, and systems outside the scope of individual groups H04L1/00 to H04L27/00).
7. A61B5 (Measurement for diagnostic purposes; Human identification).
8. G06T7 (Image analysis).
9. G06F17 (Digital computing equipment, data processing equipment, or data processing methods tailored for specific functions).
10. H04L12 (Data exchange networks).

**Figure 6:**
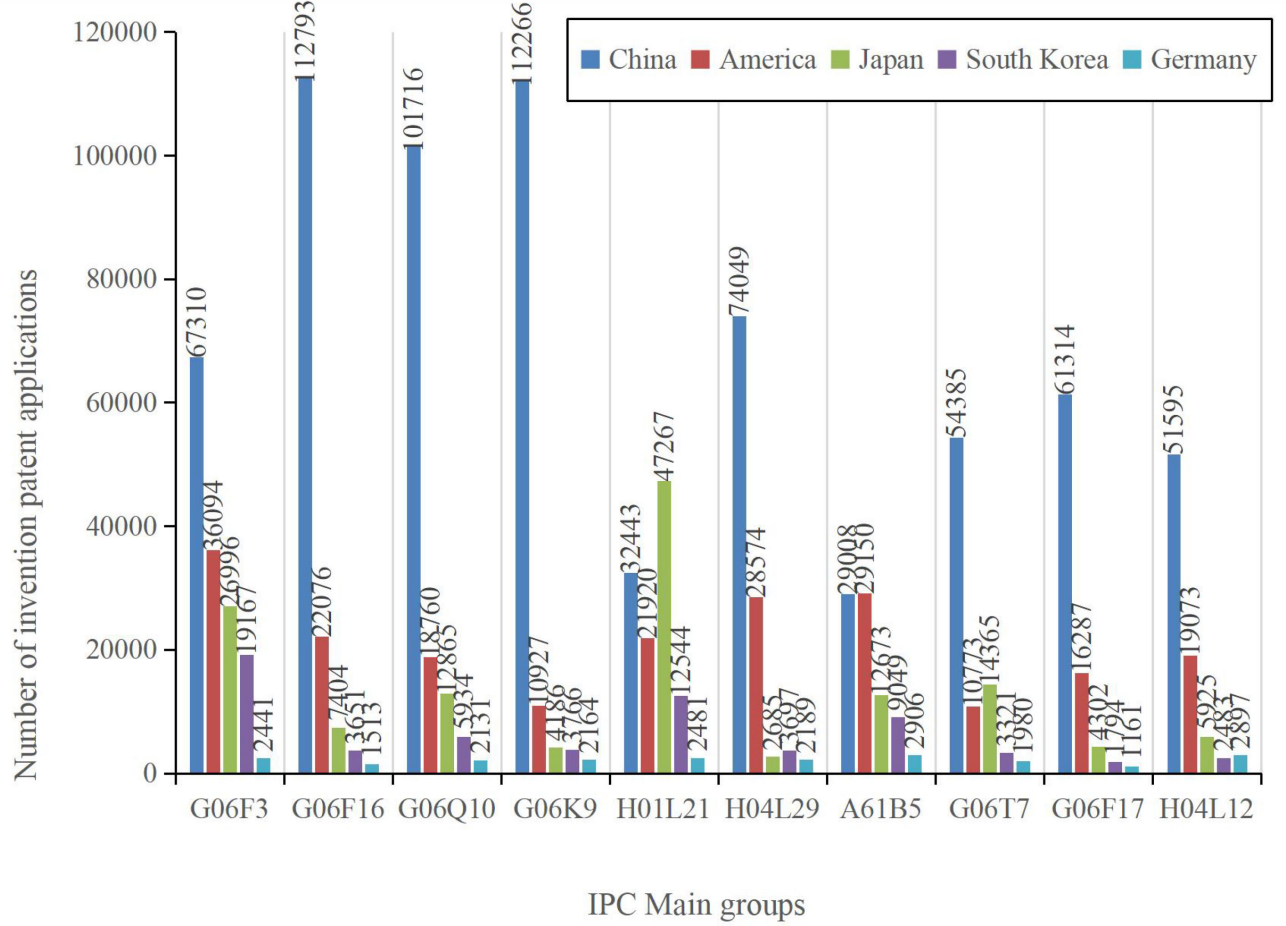
Patent technology composition of the top 5 countries in terms of the cumulative number of invention patents applied in the 224 IPC main groups of intelligent phytoprotection from 2017 to 2021.

The findings from this analysis are visualized in **Figure 6**. As depicted in **Figure 6**, it is notable that, with the exception of H01L21, China leads in the number of invention patent applications across the remaining nine patent groups. This data underscores China’s substantial contributions to innovation in the field of intelligent phytoprotection technology.

By comparing **Figures 4** and **Figure 6**, we can discern the following insights:

Among the 1,131 granted intelligent phytoprotection inventions discovered in our search results, the top five groups with the highest total patent applications for inventions were B64D1, A01M7, G05D1, A01N25, and A01C1. These patent groups predominantly encompass technologies related to drone structure design, flight control, pest trapping methods, and plant seed treatment methods.

In contrast, when examining the dataset of 3,429,252 patent applications filed for invention patents related to intelligent phytoprotection during the period from 2017 to 2021, the top 5 IPC main groups were G06F3, G06F16, G06Q10, G06K9, and H01L21. These patent groups mainly pertain to electric signal processing, computer vision, and semiconductor processing technologies. The reason for this disparity lies in the broader global applicability of the technologies within the latter category, leading to a higher volume of patents filed under this main IPC category as its application area. This divergence underscores the vital role played by these technologies in the emergence and evolution of smart plant protection technology.

**Figure 7:**
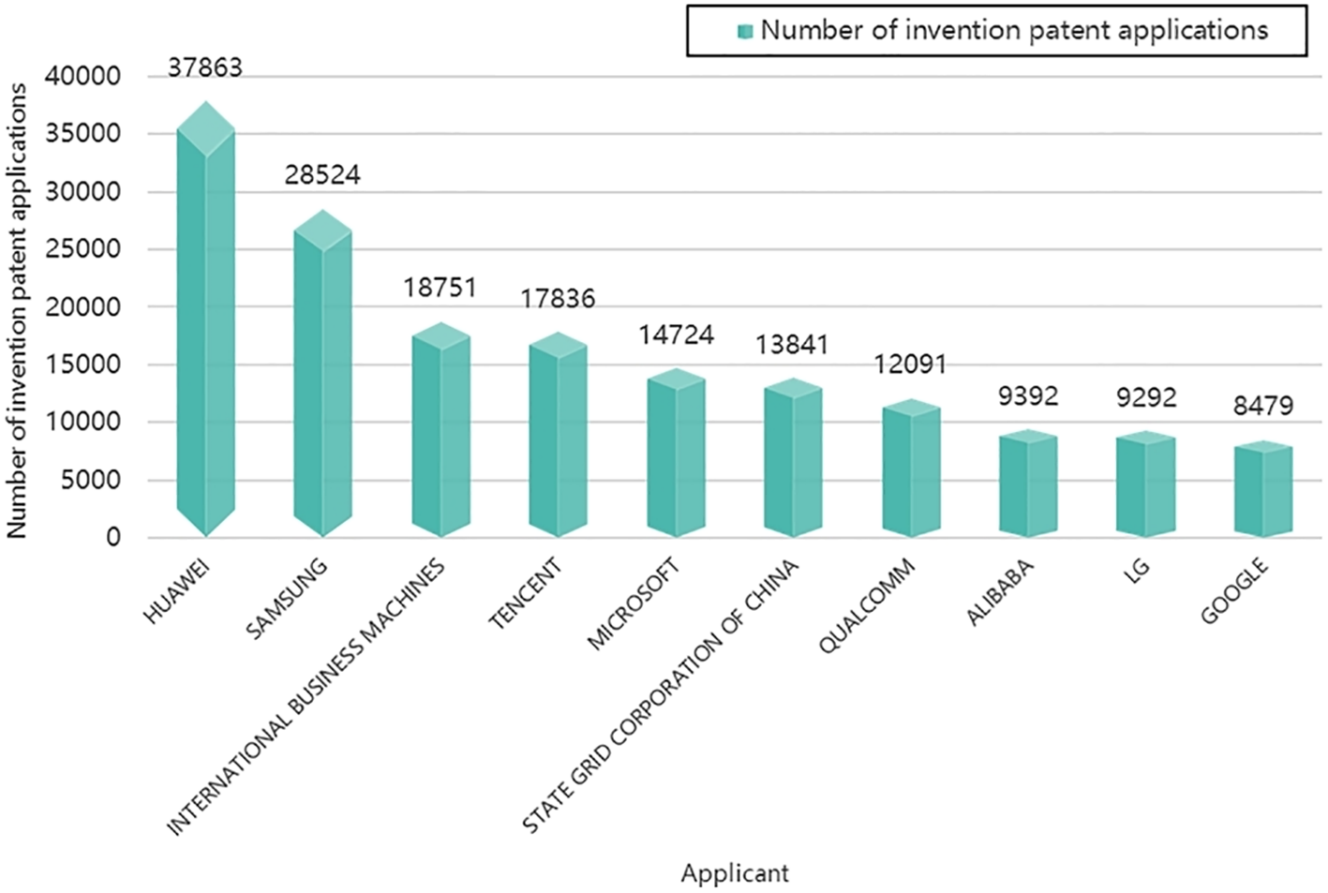
Top 10 global applicants for invention patents in the field of intelligent phytoprotection.

Furthermore, we utilized applicant information from the invention patent application records to compile a list of the top 10 worldwide applicants for invention patents. This information is presented in **Figure 7**. **Figure 7** reveals that four out of the top 10 companies are based in China, with Huawei Technologies Co., Ltd ranking at the forefront. Apart from China, the list includes four US companies (International Business Machines Corporation, Microsoft Technology Licensing LLC, Qualcomm Incorporated, Google LLC) and two South Korean companies (Samsung Electronics Co., Ltd, LG Electronics INC). This data underscores the global prominence of companies from China and other leading tech giants in shaping the landscape of intelligent phytoprotection through their significant patent activities.

The examination of the aforementioned patent data pertaining to intelligent plant protection underscores China’s current prominence in this field. To understand the factors contributing to China’s success, it is imperative to explore the role played by strategic industrial policy planning and implementation in this trajectory. Consequently, our forthcoming analysis and discussion will delve into pertinent industrial policy documents within the Chinese context.

## Industrial policy and the development of intelligent phytoprotection in China

### Chinese industrial policy

Industrial policy has a deep-rooted historical background. Dating back to the 13th century, the United Kingdom began to formulate and implement industrial policies to develop its wool textile industry. Measures included the recruitment of skilled weavers from Flanders, the imposition of tariffs on imported sheep wool products, and a range of other interventions. These industrial policies built significant competitive advantages for its textile industry before the industrial revolution in the UK (Yülek, 2018). Subsequently, in the post-Industrial Revolution era, the British government continued to implement analogous industrial policies, encompassing edicts such as forbidding the export of machinery and the emigration of skilled labor to foreign nations. Beyond the UK, the United States, France, and even the subsequently established European Union likewise embraced analogous industrial policies during the 17th to 20th centuries. For instance, the United States erected formidable tariffs, averaging approximately 40%, from the 1820s to the 1930s to shield the progression of its domestic manufacturing sector (Irwin, 2000; Harrison and Rodriguez-Clare, 2009).

In its embryonic phase, industrial policy signified an array of selective government interventions primarily targeted at the manufacturing realm. Early deployments encompassed export subsidies and tariff barriers, designed to buttress local industries’ viability while furnishing micro-entities with a practical platform for experiential learning and the accrual of technological prowess and capital. Once indigenous industries matured, and advanced production techniques surfaced, strategies shifted toward preserving the nation’s leadership within the respective technological domain by thwarting the diffusion of knowledge. Within the development economics literature, industrial policy is frequently intertwined with industrialization policy (Lall, 1987). This is vividly illustrated in the post-World War II industrial policies enacted by East Asian nations like Japan, South Korea, Singapore, and China, which encompassed measures geared toward amplifying domestic industrial technology accumulation and development, including the stimulation of exports (e.g., export tax rebates and subsidies), the attraction of foreign direct investment (primarily from multinational corporations in developed nations), the provision of credit approvals tailored for specific sectors, and the provision of skills training for the workforce (Amsden, 1989; Wade, 1990; Chang, 2009).

In the aftermath of World War II, a succession of post-war recovery and economic construction initiatives unfolded, with governmental intervention at the helm. Japan, in East Asia, experienced rapid economic ascent via its adept execution of industrial policy (Johnson, 1982), while South Korea and Taiwan astutely reshaped and modernized their indigenous industrial landscapes through meticulous industrial policy formulation and execution (Amsden, 1989; Wade, 1990; Rodrik, 1995; Amsden & Chu, 2003). For China, since 1949, industrial policies have been systematically enacted through the formulation and execution of Five-Year Plans. In its nascent stages, these policies principally centered on industry development, endeavoring to pivot from an agrarian economy to an industrialized one via the centralized allocation of resources earmarked for foundational industries such as steel and machinery manufacturing. This period, spanning from the first Five-Year Plan (1953-1957) to the eighth (1991-1995), mirrored the Soviet Union’s model, which placed hefty emphasis on heavy and chemical industry development and substantial project investments, thereby engendering extensive yet inefficacious growth approaches. With the initiation of the ninth Five-Year Plan (1996-2000), China embarked on a gradual transition away from resource-driven, investment-fueled extensive growth toward innovation-driven, high-quality, and intensive growth.

In addition to the Five-Year Plan, China began assimilating and dissecting industrial policy concepts from Japan and South Korea during the 1980s. By 1989, China had formulated its inaugural industrial policy document (Guo Fa [1989] No. 29). This document, predicated on the product characteristics of pertinent industries, delineated three categories—key support, stringent restrictions, and production proscriptions—and devised specific reference catalogs. Subsequently, China introduced a succession of industrial policies aimed at cultivating a modern industrial system and catalyzing the socialist market economy’s transformation. Notable among these were the National Industrial Policy Outline for the 1990s (Guo Fa [1994] No. 33) and the Guidance Catalogue for Industrial Structure Adjustment (in both its 2005 and 2011 editions).

As China’s economic development advanced and pertinent industrial technologies accrued, development objectives gradually shifted from mere industrialization to the nurturing of emergent industries and advanced technologies. Commencing with the 1995 proposition of the “Rejuvenating the Country through Science and Education” strategy, the Chinese government sequentially instituted a medley of industrial policies targeting strategically significant and promising industries. Spanning from the High-tech Industry initiatives of the 1990s (see Guo Fa [1991] No. 12) and the Guidance for the Key Areas of High-tech Industrialization Prioritized for Development (1999) (see Ji Gao Ji [1999] No. 827, since invalidated), to the advent of Strategic Emerging Industries in 2010 (see Guo Fa [2010] No. 32) and the unveiling of Made in China 2025 in 2015 (see Guo Fa [2015] No. 28), the Chinese government endeavored to channel industrial policies toward steering and expediting the growth of burgeoning sectors.

This analysis reveals that China’s industrial policy essentially comprises two facets: the Five-Year Plan, refreshed every quinquennial cycle, serves as the cardinal national industrial policy program, charting the course for industrial development. Complementary to this, specific industrial policies such as those pertaining to strategic emerging industries furnish supplementary elucidations of the Five-Year Plan, elucidating with finer granularity the scope, target demographic, and temporal horizons underpinning the execution of industrial policies.

Eminent scholars contend that the economic ascension of East Asian nations in the 1960s can be attributed to industrial policies featuring measures to stimulate technological accumulation and innovation capabilities, including initiatives to advance education and worker skills training (Stiglitz and Greenwald, 2014). The capacity of late-developing countries to bridge the industrial divide with their developed counterparts hinges largely on the accumulation of technological acumen and innovation capabilities in pertinent industries, as well as the fostering of an external milieu conducive to localized learning and innovation (Cimoli et al., 2009a; Cimoli et al., 2009b). Owen (2012) posits that industrial policy’s central tenet is the transformation of contemporary economic structures to drive economic development and construction, with the policy ambit naturally encompassing measures aimed at propelling technological innovation. Notably, since the advent of the 21st century, the United States has embedded a technological innovation dimension within its industrial policies. Entities like the National Science Foundation (NSF), National Aeronautics and Space Administration (NASA), and Department of Defense, whether directly or indirectly, underwrite the innovation undertakings of micro-entities like corporations, universities, and nonprofit institutions via project mandates and procurement practices, furnishing them with substantial impetus for economic development (Mazzucato, 2015).

## Characteristics analysis of Chinese industrial policies related to the development of intelligent phytoprotection

Intelligent phytoprotection, by definition, represents a convergence of traditional plant protection methods with information technology, advanced equipment manufacturing, and digital technology. Its inception and growth have been closely intertwined with the expansion of emergent industries like information technology, which, given the global drive toward industrial modernization, has garnered significant attention and robust support from national industrial policies across the globe. Among the world’s most prolific users of industrial policies, China stands out with its central government-driven Five-Year Plans, delineating precise pathways for the development of primary, secondary, and tertiary sectors (Juhász et al., 2023).

To identify the industrial policy documents shaping the development of intelligent phytoprotection in China, this study employs text analysis methods. These methods encompass searching for document names on the website of the State Council of China, meticulous review of China’s fourteen Five-Year Plan documents, and conducting word frequency analyses on the relevant documents. These techniques provide valuable insights into the core content and objectives outlined in pertinent industrial policy documents. A content scrutiny of China’s fourteen Five-Year Plans documents reveals that, commencing with the Eleventh Five-Year Plan (2006-2010), the Chinese government embarked on a mission to modernize agriculture, revamp conventional farming practices, and elevate agricultural mechanization levels (The State Council of the People’s Republic of China, 2006). Notably, agricultural informatization and related concerns did not initially feature in policy texts, owing to ample room for enhancing mechanization levels in China’s agricultural landscape. However, with the advent of the Twelfth Five-Year Plan (2011-2015), the Chinese government escalated its commitment, advocating for accelerated agricultural scientific and technological innovation, the development of agricultural information technology, and the augmentation of agricultural production and management informatization. Herein, driven by both international industry trends and domestic developmental exigencies, policy documents began emphasizing the imperative of advancing agricultural informatization. This period marked a pivotal transition from agricultural mechanization to agricultural informatization, laying the foundation for the nascent stages of smart agriculture and intelligent phytoprotection.

Upon a meticulous examination of key government documents in China, it became evident that the term “intelligent phytoprotection” was explicitly featured in a working document issued by the Ministry of Rural Affairs and Agriculture in March 2023. Consequently, the search strategy was adjusted, and “smart agriculture” was employed as a keyword, yielding 354 central documents vocally championing the cause of smart agriculture. One exemplar of this advocacy was the “Outline of the 13th Five-Year Plan for National Economic and Social Development of the People’s Republic of China,” unveiled and set in motion in March 2016. This document unambiguously articulated the necessity of fortifying the ecosystem for modern agricultural science and technology innovation, accelerating the pace of agricultural mechanization, intensifying the fusion of agriculture and information technology, nurturing smart agriculture, and enhancing agricultural productivity (The State Council of the People’s Republic of China, 2016). Similarly, the “14th Five-Year Plan for National Economic and Social Development and the Outline of Long-Term Goals for 2035,” unveiled and implemented in March 2021, underscored the need to expedite the evolution of smart agriculture and champion the digital transformation of agricultural production, operations, and management services (The State Council of the People’s Republic of China, 2021).

China’s industrial policies, typically promulgated by the State Council via programmatic documents, trigger the release of supplementary policy documents by various State Council agencies, contingent on the nature of their specific responsibilities. Scrutinizing the issuing authorities of the 354 documents, it emerged that the preeminent contributor was the Ministry of Agriculture and Rural Affairs, accounting for 111 documents. Following in the tally were the Ministry of Industry and Information Technology (44 documents), the Ministry of Agriculture (42 documents), the Ministry of Finance (27 documents), and the Ministry of Science and Technology (25 documents). In the domain of intelligent phytoprotection-related industrial policy documents, the Ministry of Agriculture and Rural Affairs and the Ministry of Industry and Information Technology lead the charge in terms of formulation and implementation. Conducting word frequency analysis on the 12th Five-Year Plan document, released concurrently with a strategic emerging industry policy document in the same year, it was found that the word “innovation” appeared 117 times, which had never occurred in previous five-year plan documents. This observation aligns with the viewpoint presented by Salahuddin and Yülek (2022), emphasizing the pivotal juncture in China’s industrial policy landscape. The year 2010 marked a critical watershed for China’s industrial policies. The issuance of two pivotal industrial policy documents by the Chinese government in 2010, encompassing the strategic emerging industry policy and the 12th Five-Year Plan, marked the shift from an emphasis on industrialization in the early stages to a more pronounced focus on promoting innovation and nurturing emerging industries (The State Council of the People’s Republic of China, 2010; 2011). This transition sought to steer structural transformations within the industrial landscape through policy interventions and enhance resource allocation to sectors yielding high-value-added outputs.

During the Twelfth Five-Year Plan era, the Chinese government introduced a pivotal national industrial policy - the Strategic Emerging Industry policy. Embodied in the “Decision of the State Council on Accelerating the Cultivation and Development of Strategic Emerging Industries” (Guo Fa [2010] No. 32 Document). It outlined the identification of seven key sectors as nationally strategic emerging industries deserving robust support. These sectors encompassed Biotechnology, New Generation Information Technology, High-end Equipment Manufacturing, New Materials, Energy Conservation and Environmental Protection, New Energy, and New Energy Vehicles (The State Council of the People’s Republic of China, 2010). This strategic decision had a cascading effect, propelling the advancement of technologies that form the foundation of intelligent phytoprotection. This included advancements in drone manufacturing, artificial intelligence, big data analytics, and other related fields. These technological strides were instrumental in shaping and advancing the landscape of intelligent phytoprotection in China.

According to the analysis of patent data in the previous section, it can be found that the technological field of intelligent phytoprotection has a great intersection with the new generation of information technology industry and high-end equipment manufacturing industry, both of which are strategic emerging industries proposed by China in 2010. Therefore, the implementation of strategic emerging industry policies may have an important impact on intelligent phytoprotection innovation activities. Firstly, the government, through the planning and implementation of strategic emerging industry policies, has listed strategic emerging industries such as the new generation of information technology industry and high-end equipment manufacturing industry, which have high technological intersection with intelligent phytoprotection, as key areas worthy of support and cultivation. At the policy level, it has clarified the importance and development direction of intelligent phytoprotection innovation activities, providing macro guidance and policy support for their rapid development. Secondly, strategic emerging industry policies provide financial support and talent training guarantees for intelligent phytoprotection innovation activities. The government, through the establishment of special funds and the provision of financial subsidies, provides necessary research and development funds and equipment procurement funds for intelligent phytoprotection innovation activities, promoting their technological progress and innovation ability. Although these policies may not be directly named in the name of intelligent phytoprotection, the technical fields (such as artificial intelligence, agricultural machinery equipment manufacturing, computer vision processing, 3S technology, etc.) supported by the research and development activities have a high correlation with intelligent phytoprotection. Thirdly, the planning and implementation of strategic emerging industry policies provide opportunities for market promotion and application of intelligent phytoprotection innovation activities. The government has clearly designated strategic emerging industries such as the digital economy industry as national-level strategic emerging industries, which not only promotes the development of the digital economy, but also provides more opportunities and resources for the market promotion of intelligent phytoprotection innovation activities.

## Research on the impact of industrial policy on the innovative activities of intelligent phytoprotection

### Methodology and data

Intelligent phytoprotection encompasses technologies associated with information technology sectors such as sensor technology, network communication technology, and artificial intelligence. Additionally, it encompasses technologies linked to equipment manufacturing industries, including unmanned aerial vehicle manufacturing, as well as digital creative industries, such as digital facility planting, digital forestry, and automated breeding. Notably, all three of these industries were classified as strategic emerging industries by the Chinese government by 2010. Consequently, this study amalgamates the analysis of industrial policies outlined in the preceding section and designates the year 2010, when the 12th Five-Year Plan and the Strategic Emerging Industry policies were formulated and enacted, as the year in which these industrial policies came into effect. In theoretical terms, the influence of industrial policies on patent applications within the realm of intelligent phytoprotection technology would commence in 2011.

We compiled a list of 224 IPC main groups, leveraging IPC main technology classification data from 1131 granted invention patents related to intelligent phytoprotection, garnered through patent retrieval and analysis. These 224 main groups constitute the candidate pool for treatment groups affected by the policy. Concurrently, we excluded major patent groups that did not fall under the 224 IPC main groups for intelligent phytoprotection and those associated with strategic emerging industries within the International Patent Classification (IPC 2020.01). The remaining IPC main groups constituted the alternative pools for the control group. Furthermore, we harnessed the technology inclusion relationships spanning various technology classification levels within the International Patent Classification System, encompassing Section-Class-Subclass-Main group-Subgroup, to pair treatment and control groups belonging to the same IPC subclass. This method allowed us to mitigate estimation errors attributable to technological disparities across different IPC main groups. Subsequently, prior to commencing regression estimation, we identified 143 IPC main groups for the treatment group and 811 for the control group.

To facilitate our research, we utilized the comprehensive dataset of Chinese invention patent applications retrieved from the incoPat database, spanning the years 2005 to 2020 and comprising a total of 10,408,297 applications. We designated the year 2010 as the policy year. With this dataset in hand, the study constructed a Difference-in-Difference (DID) model to assess the relationship between industrial policies and patent applications within the domain of intelligent phytoprotection technology. Equation (1) illustrates the estimated model.


(1) $$IP_{it}=\beta_{0}+\beta_{1}\times Treat\times post_{t}+IPCsubclass_{t-1}+\delta_{year}+\gamma_{ipcgroup}+\varepsilon_{it}$$

The variable “Treat” is indicative of the number of patent applications for inventions related to intelligent phytoprotection at the IPC main group level. In cases where a patent’s IPC main group belongs to the 224 categories identified as intelligent phytoprotection, “Treat” is assigned a value of 1; otherwise, it is set to 0. “Post” is designated as the year 2010, signifying the year when the policy impact took effect. Subsequent years are coded as 1. “Pre” represents the number of patent applications for collaborative innovation and invention at the IPC subclass level in the year t-1, serving as a control variable to account for the growth trend of each patent group over time and to mitigate any inherent issues arising from missing variables. “Year” and “IPC main group” symbolize the fixed effects associated with the year and the IPC main group, respectively. It is noteworthy that these fixed effects have been incorporated into equation (1), obviating the need for separately including the “Treat” and “Post” variables to avoid multicollinearity issues. Lastly, “ϵ” denotes the random disturbance term. The data processing and regression estimations conducted in this study were executed using Stata 16.0.

### Empirical results

**Figure 8:**
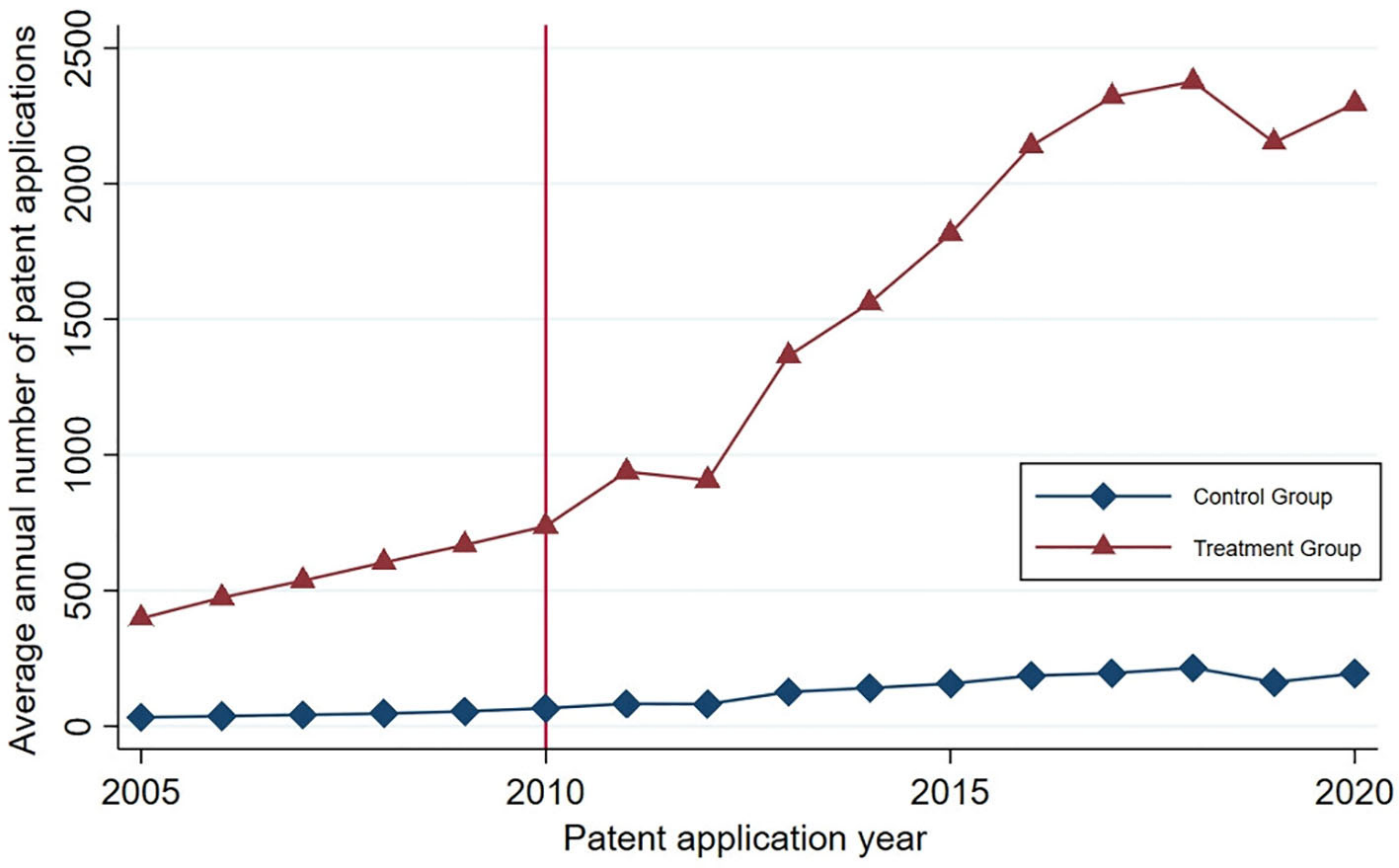
The average annual number of invention patents applied by the treatment group and the control group before and after the implementation of industrial policies.

Initially, we computed the average annual patent applications for inventions within the treatment group and the control group from 2005 to 2020. We then represented these averages in **Figure 8**, employing a triangular icon to signify the treatment group and a square icon for the control group. As depicted in **Figure 8**, although the treatment group exhibited higher absolute average annual patent applications for inventions compared to the control group, they both displayed a similar growth trajectory until 2010. Post-2010, the treatment group’s growth rate in invention patent applications gradually outpaced that of the control group. This outcome suggests that the series of industrial policies enacted since 2010 may have fostered an increase in invention patent applications within the treatment group.

Subsequently, we assessed the relationship between industrial policies and patent application activities in the technical domains related to intelligent phytoprotection, employing equation (1). The regression results are presented in **Table 1**. The second column of the regression results, which includes control variables, demonstrates that industrial policy exerted a significantly positive influence on the activities involving invention patent applications for intelligent phytoprotection technology. Following the policy implementation, the treatment group exhibited an average increase of approximately 1131 patents in invention patent applications for intelligent phytoprotection technology when compared to the control group. This effect reached statistical significance at the 1% significance level.

**Table 1:** Estimate of the impact of industrial policy implementation on the patent application activities of technology inventions related to intelligent phytoprotection.

Variable	Dependent Variable: The application number of invention patents for intelligent phytoprotection	
	(1)	(2)
Treat*Post	1135.322^***^	1131.639^***^
	(70.732)	(75.030)
IPCsubclass_t-1_		0.001^***^
		(0.000)
IPC_maingroup Fixed Effect	YES	YES
Year Fixed Effect	YES	YES
Adjusted R^2^	0.6729	0.6932
Observations	14576	13665

^*^p<0.05, ^**^ p<0.01, ^***^p<0.001.

The number of invention patent applications serves as an indicator of the attention dedicated by micro-level entities such as enterprises, research institutes, and individuals to a particular industry and its associated technological domains. The estimated results in **Table 1** reveal that the industrial policies issued and implemented by China in 2010, particularly the 12th Five-Year Plan and the Strategic Emerging Industries Policy, directed societal innovation entities and resources towards concentrating on the technical realm of intelligent phytoprotection. This had a substantial and potent impact on the application activities related to invention patents in the field of intelligent phytoprotection within the treatment group.

According to the regression estimation results in **Table 1**, it can be found that the strategic emerging industry policy implemented by China in 2010 greatly promoted innovation activities in the field of intelligent phytoprotection, which is manifested in a significant increase in invention patent applications in the large group of intelligent phytoprotection-related patents. When assessing the extent of this influence, as indicated by the Adjusted-R2 value in column (2) of **Table 1**, it becomes evident that industrial policies can account for approximately 69.32% of the variance in the rise of innovation activities related to intelligent phytoprotection. This article adopts a methodological approach that leverages the technical similarity within international patent technology classifications at the same level to establish both the treatment and control groups based on IPC Subclass. By scrutinizing these groups from the perspective of patent technology classification, it is reasonable to conclude that, provided the trends in patent subcategories of the treatment and control groups are effectively controlled in Model (1), and with the inclusion of IPC main groups and Year as fixed effects, the estimation method employed herein is adept at isolating the distinctive influence of industrial policies on innovation activities in the field of intelligent phytoprotection, effectively mitigating the influence of other external factors.

To validate the robustness of these estimated results, this study adopted the Synthetic Difference-in-Differences (SDID) estimation method, following the approach of Arkhangelsky et al. (2021), for re-estimation based on Model 1. In comparison to the outcomes obtained through traditional DID estimation methods in **Table 1**, SDID estimation, by leveraging a re-weighting approach to match pre-processing trends and incorporate temporal weightings, demonstrated the ability to mitigate reliance on parallel trends and exclude periods significantly differing from the processing period. Moreover, SDID reduced estimation bias and enhanced accuracy.

The results presented in **Table 2** reveal that both the estimated results in column (1) without control variables and those in column (2) with control variables demonstrate a positive and statistically significant impact of industrial policies on patent application activities in the domain of intelligent phytoprotection-related technology inventions. Notably, the estimated coefficients and standard deviations of “Treat * Post” in **Table 2** are more conservative than those in **Table 1**, yet the statistical significance remains unchanged. These findings highlight the greater robustness and reliability of SDID estimation compared to traditional DID methods.

**Table 2:** Estimation results based on SDID method.

Variable	SDID	
	(1)	(2)
Treat*Post	517.680^***^	517.812^***^
Standard Error	(10.609)	(10.648)

^*^p<0.05, ^**^p<0.01, ^***^p<0.001; 95% CIs and p-values are based on Large-Sample approximations. Refer to Arkhangelsky et al. (2021) for theoretical derivations.

**Figure 9:**
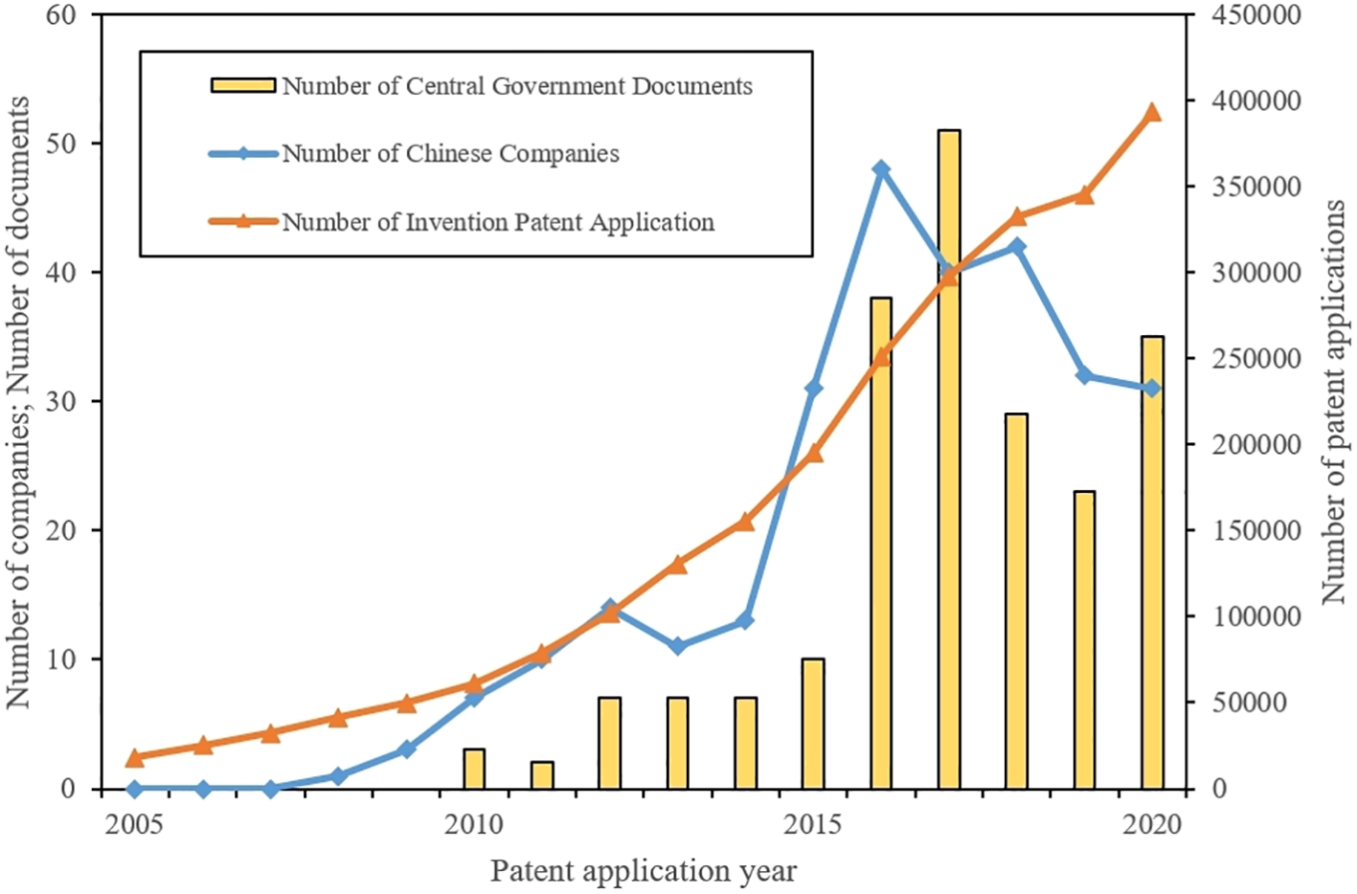
The relationship between industrial policies and invention patent applications in the field of intelligent plant protection technology. The unit value of the Number of Invention Patent Application in Intelligent Phytoprotection Technologies is measured by the right Y axis. The unit value of the Number of Chinese Listed Companies Supported by Intelligent Phytoprotection Related Industrial Policies and the Number of Central Government Documents Related to Intelligent Phytoprotection Technologies are measured by the left Y axis.

To delve further into the influence of industrial policies on the number of invention patent applications in the field of intelligent phytoprotection, we employed the 224 IPC main groups identified in the preceding section. We retrieved and identified 2,510,517 invention patent applications related to intelligent phytoprotection from the incoPat patent database. These applications were filed by Chinese entities between 2005 and 2020. Subsequently, text analysis and matching were conducted on government subsidy data obtained from the CSMAR database, focusing on Chinese listed companies. This yielded records of 283 listed companies that received government subsidies between 2005 and 2020 due to concepts related to intelligent phytoprotection, serving as a measure of the tangible impact of industrial policies at the enterprise level. In addition, we retrieved, reviewed, and statistically analyzed a total of 212 documents from 2005 to 2020 sourced from the central government’s website. These documents either contained references to Smart Agriculture in the full text or featured Strategic Emerging Industries in their titles. Finally, we presented the three sets of data in the form of a combined chart, as illustrated in **Figure 9**. **Figure 9** underscores that the implementation of industrial policies, both at the central government level and within the enterprise sphere represented by listed companies, exhibits a positive correlation with the overall trend of invention patent applications in the field of intelligent phytoprotection technology over time.

## Actionable recommendations on further promoting the development of intelligent phytoprotection

In preceding sections, we meticulously scrutinized the technological facets of intelligent crop protection utilizing global patent data, elucidating China’s eminent stature in this realm, both in terms of authorizations and applications. We posited that China’s vigorous ascendancy in intellectual property rights for crop protection could be accredited to the meticulous blueprinting and execution of pertinent industrial policies. Subsequently, our rigorous examination, which seamlessly interwove industry policy documents and patent data, effectively corroborated this assertion. Our research discerned that China’s industrial policy implementation distinctly bolstered the surge in patent applications pertaining to the advancement of intelligent phytoprotection technologies, thus catapulting China into a premier global position in the arena of intelligent phytoprotection.

Drawing from the analyses undertaken in this study, we posit that this deduction bears significant implications for propelling the evolution of intelligent phytoprotection across diverse regions worldwide. For governments in regions seeking to cultivate indigenous innovation in intelligent phytoprotection, industrial policy emerges as an indispensable policy instrument adept at orchestrating societal resources towards targeted industries. Taking a leaf from China’s industrial policy playbook, the central government commences by issuing an overarching policy blueprint, exemplified by the Five-Year Plan, which sets the stage for societal-level resource allocation expectations. Subsequently, various functional departments roll out specific operational directives, such as competitive subsidy measures, steered by professional technical agencies. These initiatives, often implemented via specialized industrial policy tools like competitive subsidies, channel societal resources into the sphere of intelligent phytoprotection, achieving the overarching objective of catalyzing innovative activities within related technology domains.

For enterprises, especially those endeavoring to establish intelligent phytoprotection-related enterprises within China, diligent attention to the Chinese government’s industrial policy documents aimed at fostering intelligent phytoprotection is advised. These documents can serve as compasses for defining research and development trajectories, guiding enterprises towards the technical domains that enjoy robust support within the purview of relevant policy documents. As an illustrative example, the 2021 Statistical Classification of Digital Economy and Its Core Industries, issued by the National Bureau of Statistics of China, encompasses intelligent agriculture categories, including Digital Facilities Planting, Digital Forestry, Automated Breeding, New Technology Breeding, and Other Intelligent Agriculture, all propelled by modern information technologies like IoT, big data, and the internet. Furthermore, the Ministry of Commerce and the Ministry of Industry and Information Technology jointly issued guidelines for international cooperation in digital economy investments, emphasizing priority support and encouragement for these domains.

**Figure 10:**
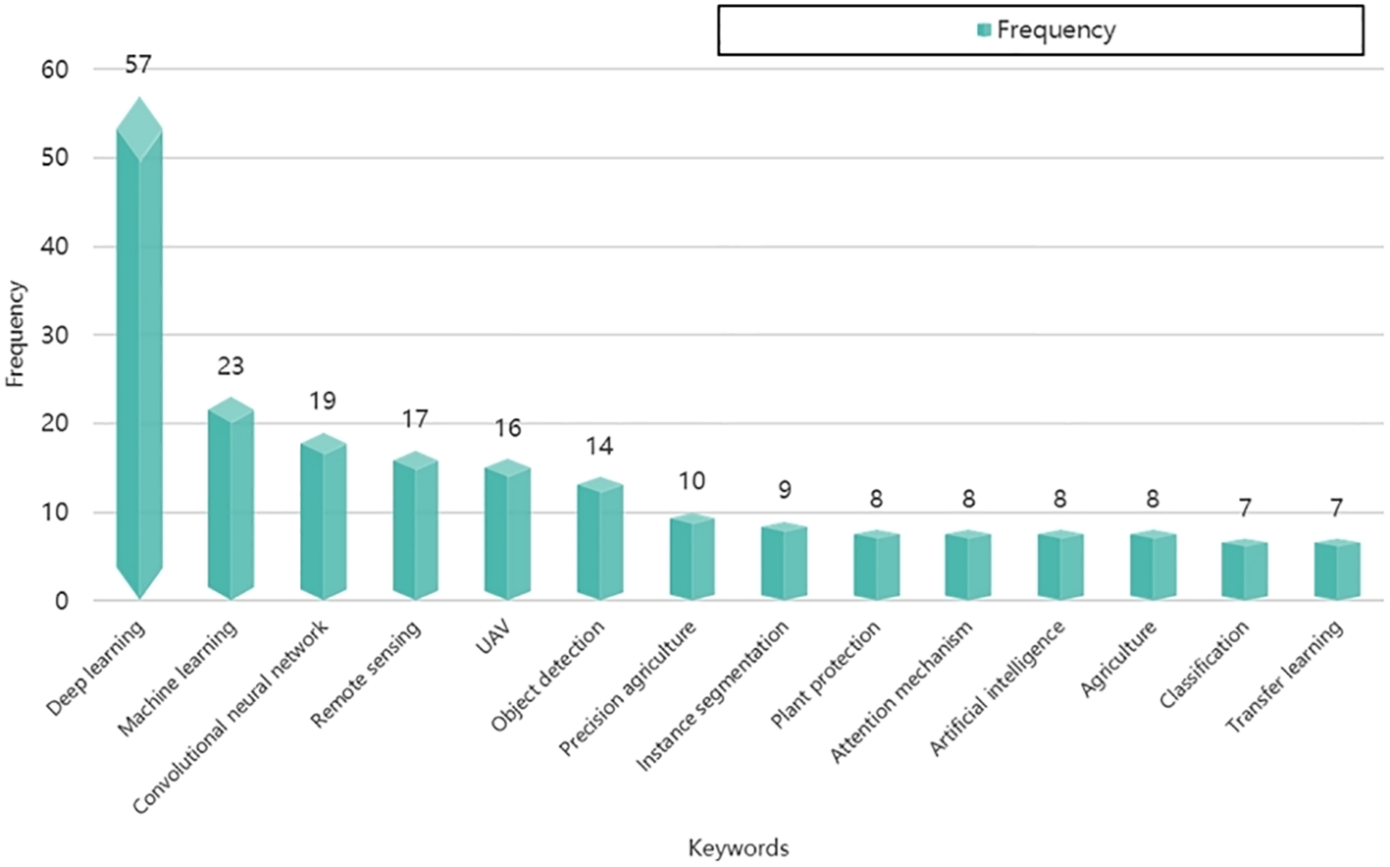
Keywords frequency analysis of the 356 articles published in the Sustainable and Intelligent Phytoprotection Section of the Frontiers in Plant Science. Note: The paper data statistics ended on August 15th, 2023.

For scholars engaged in pertinent research, an understanding of the prevailing landscape of industrial policies and patent-driven innovation activities is paramount for guiding research trajectories. In our analysis, we conducted a keyword frequency examination of 356 published papers in the Sustainable and Intelligent Phytoprotection Section of the Frontiers in Plant Science journal. Subsequently, we compiled a roster of the ten most frequently occurring keywords and presented them in **Figure 10**. The depicted figure reveals that scholars within the ambit of intelligent phytoprotection predominantly gravitate towards subjects like artificial intelligence (encompassing deep learning, machine learning, convolutional neural networks, classification algorithms, and transfer learning), remote sensing technology, computer vision technology, and unmanned aerial vehicle technology. These focal areas of research align cohesively with the insights gleaned from our prior analysis of the attributes of intelligent phytoprotection patents. Nevertheless, there exists ample room to broaden research dimensions by heeding the guidance embedded in industrial policies. For instance, intelligent Solar Insecticidal Lamp (SIL), representing an efficient and cost-effective facet of intelligent phytoprotection (Huang et al., 2023), offers promising practical utility but remains underrepresented, with a sole appearance in the keyword statistics. Scrutiny of industrial policy texts revealed that the Jiangsu Provincial Government in China explicitly featured intelligent insecticidal lamps among the recommended optional devices within a 2022 policy document titled the Guidelines for the Construction of Intelligent Multi-span Greenhouses. The incorporation of industrial policies can enhance the impetus for smart plant protection development and optimize the practical applicability of research findings.

## Discussion

This study delves into the realm of intelligent phytoprotection technology through an exhaustive analysis of patent data and the International Patent Classification System, identifying 224 IPC main groups pertinent to this field. By means of meticulous data analysis and regression estimations, the research probes the influence of industrial policies on invention patent applications within the ambit of intelligent phytoprotection technology. In contrast to conventional economic theories that often cast doubt on the efficacy of industrial policies, the empirical evidence presented herein distinctly showcases the substantial impact of industrial policy implementation on invention patent application activities within the sphere of intelligent phytoprotection technology.

As China continues to burgeon in terms of economic magnitude and technological innovation prowess, the insights garnered from China’s developmental trajectory hold relevance and resonance for other nations and regions. The study charts a viable course of action for countries and regions aspiring to cultivate technologies associated with intelligent phytoprotection. This roadmap underscores the pivotal role of judiciously planned and formulated industrial policies in catalyzing the rapid advancement of intelligent phytoprotection technology. The analysis of pertinent industrial policy documents in China conducted in this study brings to the fore the central government’s ambition to realize agricultural informatization, encompassing both smart agriculture and intelligent phytoprotection, alongside the impetus to foster the development of strategic emerging industries, notably artificial intelligence and big data.

The emergence and development of intelligent phytoprotection are essentially products of the industrialization process. Referring to Yülek (2018) viewpoint, the industrialization of a country or region can be divided into four stages: the first stage is the import and use of machinery, through which industrial knowledge can be accumulated through import and OEM. The second stage is technology adoption or technology absorption, which further realizes the digestion and absorption of industrial knowledge and promotes the accumulation of capabilities of local industrial enterprises. The third stage is imitation, through which imported products are copied or improved by combining localized knowledge to achieve the introduction-digestion-imitation of external knowledge and promote the development of local related industries. The fourth stage is innovation and product development, which gradually realizes the localization and autonomy of related industrial technologies, and then becomes the explorer and innovator in the field. Based on the analysis and discussion in the previous chapters of this article, it can be found that since 2010, the goal of China’s industrial policy has shifted to the fourth stage of industrialization - innovation and product development. According to Yülek (2018) suggestion, industrial policy needs to start transitioning to innovation policy at this time. Furthermore, it’s noteworthy that central policy documents of paramount importance for the development of smart agriculture and intelligent phytoprotection were collectively led by the Ministry of Rural and Rural Affairs of the People’s Republic of China and the Ministry of Industry and Information Technology, with the active involvement of various other relevant departments of the State Council. This cooperative orchestration and leadership helmed by specialized technical officials and their respective departments emerge as a linchpin of China’s success in leveraging industrial policies to nurture technologies pertaining to intelligent phytoprotection.

In conclusion, this research offers a compelling case for the potency of industrial policies in propelling innovation and technological advancement, notably within the sphere of intelligent phytoprotection technology. The detailed examination of China’s experiences in this context serves as a valuable reference for other countries and regions aiming to follow a similar path of development. It highlights the transformative power of well-crafted industrial policies when implemented with a clear vision and expert management.

This article specifically examines the influence of industrial policies on the volume of invention patent applications within the realm of intelligent phytoprotection in China. It is important to emphasize that the conclusions drawn in this study require some clarification. The assertion that industrial policies can drive innovation in the field of intelligent phytoprotection is context-dependent. This strategy is likely to be most effective in countries or regions that share a similar industrial foundation with China and are actively considering the adoption of industrial policies to bolster the growth of the intelligent phytoprotection industry and encourage advancements in related technology.

## Author contributions

YZ: Conceptualization, Data curation, Investigation, Software, Writing – original draft. SL: Formal Analysis, Methodology, Project administration, Supervision, Validation, Writing – review & editing. FW: Funding acquisition, Resources, Visualization, Writing – review & editing.

## References

[B1] Abbasi M. Yaghmaee M. H. Rahnama F. (2019). “Internet of Things in agriculture: A survey,” in Proceedings Of 2019 3rd International Conference On Internet Of Things And Applications (IoT) Isfahan, Iran. 1–12. doi: 10.1109/IICITA.2019.8808839

[B2] Amsden A. (1989). Asia’s next giant: South Korea and late industrialization[M] (New York: Oxford University Press).

[B3] Amsden A. Chu W. W. (2003). Beyond late development: Taiwan’s upgrading policies [M] (Cambridge, MA: The MIT Press).

[B4] Arkhangelsky D. Athey S. Hirshberg D. A. Imbens G. W. Wager S. (2021). Synthetic difference-in-differences. Am. Econ. Rev. 111 (12), 4088–4118. doi: 10.1257/aer.20190159

[B5] Avsar E. Bulus K. Saridas M. A. Kapur B. (2021). Evaluation of an electronic irrigation system with internet connection in strawberry cultivation. Environ. Eng. Manage. J. 20 (9), 1487–1497. doi: 10.30638/eemj.2021.138

[B6] Benos L. Tagarakis A. C. Dolias G. Berruto R. Kateris D. Bochtis D. (2021). Machine learning in agriculture: A comprehensive updated review. Sensors 21 (11), 3758. doi: 10.3390/s21113758 34071553 PMC8198852

[B7] Chang H. J. (2009). “Industrial policy: can we go beyond an unproductive confrontation?” in Paper presented at the 2009 World Bank ABCDE Conference(22-24 June, Seoul).

[B8] Chin R. Catal C. Kassahun A. (2023). Plant disease detection using drones in precision agriculture. Precis. Agric. 24 (5), 1663–1682. doi: 10.1007/s11119-023-10014-y

[B9] Cimoli M. Dosi G. Nelson R. R. Stiglitz J. (2009a). “Institutions and policies shaping industrial development: an introductory note.” Industrial Policy and Development: The Political Economy of Capabilities Accumulation, Initiative for Policy Dialogue. Eds. Cimoli M. Dosi G. J. Stiglitz E. (Oxford, UK: Oxford University Press), 19–38. doi: 10.1093/acprof:oso/9780199235261.003.0002

[B10] Cimoli M. Dosi G. Nelson R. R. Stiglitz J. (2009b). “The Political Economy of Capabilities Accumulation: The Past and Future of Policies for Industrial Development.” Industrial Policy and Development: The Political Economy of Capabilities Accumulation, Initiative for Policy Dialogue. Eds. Cimoli M. Dosi G. J. Stiglitz E. (Oxford, UK: Oxford University Press), 1–16.

[B11] European Commission. (2012). A stronger european industry for growth and economic recovery (Brussels: Industrial Policy Communication Update COM(2012) 582 final). Available at: https://www.eesc.europa.eu/en/our-work/opinions-information-reports/opinions/stronger-european-industry-growth-and-economic-recovery-industrial-policy-communication-update-com2012-582-final#downloads.

[B12] Harrison A. E. Rodriguez-Clare A. (2009). Trade, foreign investment, and industrial policy[R] (Munich: University Library of Munich). MPRA Paper 15561.

[B13] Huang K. Shu L. (2021). Grand challenges in sustainable and intelligent phytoprotection. Front. Plant Sci. 12, 2513. doi: 10.3389/fpls.2021.755510 PMC8635507 34868144

[B14] Huang K. Shu L. Li K. L. Chen Y. J. Zhu Y. Valluru R. (2023). Sustainable and intelligent phytoprotection in photovoltaic agriculture: new challenges and opportunities. Electronics 12 (5), 1221. doi: 10.3390/electronics12051221

[B15] Irwin D. (2000). Did late-nineteenth-century U.S. Tariffs promote infant industries? Evidence from the tinplate industry. J. Economic History 60 (2), 335–360. doi: 10.1017/S0022050700025122

[B16] Johnson C. (1982). MITI and the Japanese miracle: the growth of industrial policy: 1925-1975 [M.] (Stanford, California: Stanford University Press).

[B17] Juhász R. Lane N. Rodrik D. (2023) The new economics of industrial policy. Available at: https://tinyurl.com/2ckdgn4z.

[B18] Kumar S. (2020). Technological intercropping with the cloud, ioT, and big data in Indian organic agriculture. Int. Manage. Rev. 16 (2), 94–104.

[B19] Lall S. (1987). Learning to industrialize[M] (London: Macmillan).

[B20] Li D. (2021). Application of artificial intelligence and machine learning based on big data analysis in sustainable agriculture. Acta Agriculturae Scandinavica: Section B. Soil Plant Sci. 71 (9), 956–969. doi: 10.1080/09064710.2021.1965650

[B21] Mazzucato M. (2015). The entrepreneurial state: debunking public vs. Private sector myths[M] (New York: Public Affairs).

[B22] Mohan S. S. Venkat R. Rahaman S. Vinayak M. Babu B. H. (2023). Role of AI in agriculture: applications, limitations and challenges: A review. Agric. Rev. 44 (2), 231–237. doi: 10.18805/ag.R-2215

[B23] Naudé W. (2010). Industrial policy: old and new issues[R]. Working paper no.2010/106 (United Nations University: World Institute for Development Economics Research).

[B24] Oppenheim D. Shani G. Erlich O. Tsror L. (2019). Using deep learning for image-based potato tuber disease detection. Phytopathology 109 (6), 1083–1087. doi: 10.1094/PHYTO-08-18-0288-R 30543489

[B25] Owen G. (2012). Industrial policy in europe since the second world war: what has been learnt, ECIPE Occasional Paper, No.1 (Brussels, Belgium: The European Centre for International Political Economy).

[B26] Phasinam K. Kassanuk T. Shabaz M. (2022a). Applicability of internet of things in smart farming. J. Food Qual. 2022, 7692922. doi: 10.1155/2022/7692922

[B27] Phasinam K. Kassanuk T. Shinde P. P. Thakar C. M. Sharma D. K. Mohiddin M. K.. (2022b). Application of ioT and cloud computing in automation of agriculture irrigation. J. Food Qual. 2022, 8285969. doi: 10.1155/2022/8285969

[B28] Qin F. Liu D. X. Sun B. D. Ruan L. Ma Z. H. Wang H. G. (2016). Identification of alfalfa leaf diseases using image recognition technology. PloS One 11 (12), e0168274. doi: 10.1371/journal.pone.0168274 27977767 PMC5158033

[B29] Rao Z. Yuan J. (2021). Data mining and statistics issues of precision and intelligent agriculture based on big data analysis[J]. Acta Agriculturae Scandinavica: Section B. Soil Plant Sci. 71 (9), 870–883. doi: 10.1080/09064710.2021.1954684

[B30] Rathod M. L. Shivaputra A. Umadevi H. Nagamani K. Periyasamy S. (2022). Cloud computing and networking for smart farm agri tech. J. Nanomaterials 2022, 6491747. doi: 10.1155/2022/6491747

[B31] Rodrik D. (1995). Getting interventions right: how South Korea and Taiwan grew rich[J]. Economic Policy 10 (20), 55–107. doi: 10.2307/1344538

[B32] Roshan S. H. Kazemitabar J. Kheradmandian G. (2022). Artificial intelligence aided agricultural sensors for plant frostbite protection. Appl. Artif. Intell. 36 (1), 1–24. doi: 10.1080/08839514.2022.2031814

[B33] Salahuddin T. Yülek M. A. (2022). Structural breaks, manufacturing revolutions, and economic catch-up: empirical validation of historical evidence from South Korea. J. Asian Finance Economics Business 9 (1), 13–24. doi: 10.13106/jafeb.2022.vol9.no1.0013

[B34] Singh P. Verma A. Alex J. S. R. (2021). Disease and pest infection detection in coconut tree through deep learning techniques[J]. Comput. Electron. Agric. 182, 105986. doi: 10.1016/j.compag.2021.105986

[B35] Sishodia R. P. Ray R. L. Singh S. K. (2020). Applications of remote sensing in precision agriculture: A review. Remote Sens. 12 (19), 3136. doi: 10.3390/rs12193136

[B36] Stiglitz J. E. (2017). “Industrial policy, learning, and development,” in The practice of industrial policy: government-business coordination in africa and east asia. Ed. Tarp F.. (Oxford, UK: Oxford University Press).

[B37] Stiglitz J. E. Greenwald B. C. N. (2014). Creating a learning society: A new approach to growth, development, and social progress[M]. (New York: Columbia University Press).

[B38] The Ministry of Economy, Trade, and Industry of Japan (2010). Industrial structure vision 2010 (Tokyo: Ministry of Economy, Trade and Industry).

[B39] The State Council of the People’s Republic of China. (2006). The 11th Five-year plan of national economy and social development of the People’s Republic of China. (Beijing: The State Council of the People's Republic of China).

[B40] The State Council of the People’s Republic of China. (2010). The decision of the state council on accelerating the cultivation and development of strategic emerging industries. (Beijing: The State Council of the People's Republic of China).

[B41] The State Council of the People’s Republic of China. (2011). The 12th Five-year plan of national economy and social development of the People’s Republic of China. (Beijing: The State Council of the People's Republic of China).

[B42] The State Council of the People’s Republic of China. (2016). The 13th Five-year plan of national economy and social development of the People’s Republic of China. (Beijing: The State Council of the People's Republic of China).

[B43] The State Council of the People’s Republic of China. (2021). The 14th five year plan for national economic and social development of the people’s republic of China and the outline of the long range goals for 2035. (Beijing: The State Council of the People's Republic of China).

[B44] Wade R. (1990). Governing the market: economic theory and the role of government in east asian capitalism[M] (Princeton, NJ: Princeton University Press).

[B45] Wang H. (2022). Smart phytoprotection and suggestions for its development. J. China Agric. Univ. 27, 1–21. doi: 10.11841/j.issn.1007-4333.2022.10.01

[B46] Wang Z. G. Wang H. Q. Feng M. C. Qin M. X. Zhang X. R. Xie Y. K.. (2021). Study on the monitoring and classification of winter wheat freezing injury in spring based on 3S technology. J. Agric. Sci. 159 (9/10), 623–635. doi: 10.1017/S0021859621001076

[B47] Yülek M. A. (2018). How nations succeed: manufacturing, trade, industrial policy, & Economic development [M]. (New York: Palgrave Macmillan US).

